# Probable disseminated *Mycobacterium abscessus* subspecies *bolletii* infection in a patient with idiopathic CD4+ T lymphocytopenia: a case report

**DOI:** 10.1186/1752-1947-6-277

**Published:** 2012-09-04

**Authors:** Claudia Colomba, Raffaella Rubino, Paola Di Carlo, Caterina Mammina, Celestino Bonura, Lucia Siracusa, Lucina Titone, Laura Saporito

**Affiliations:** 1 Dipartimento di Scienze per la Promozione della Salute, Università di Palermo, Via del Vespro 129, Palermo, 90127, Italy; 2 Dipartimento di Scienze per la Promozione della Salute, Sezione di Malattie Infettive, Università di Palermo, Via del Vespro 129, Palermo, 90127, Italy

**Keywords:** CD4+ T lymphocytopenia, Clarithromycin, Disseminated infection, *Mycobacterium abscessus* subsp. *bolletii* rapidly growing mycobacteria

## Abstract

**Introduction:**

Rapidly growing mycobacteria are opportunistic pathogens in patients with underlying risk factors. *Mycobacterium abscessus* subsp. *bolletii* is a newly recognized member of rapidly growing mycobacteria, isolated from respiratory tract and cutaneous infections.

**Case presentation:**

We describe a case of chronic disseminated infection caused by *M. abscessus* subsp. *bolletii* in a 38-year-old Sri Lankan man with idiopathic CD4+ T lymphocytopenia. Idiopathic CD4+ T lymphocytopenia is a rare cause of immunodysfunction that, similar to human immunodeficiency virus infection, causes a depletion of CD4+ T lymphocytes. *M. abscessus* subsp. *bolletii* infection was diagnosed by culture isolation from two sputum samples.

**Conclusions:**

To the best of our knowledge this is the first report of *M. abscessus* subsp. *bolletii* disseminated infection in a patient affected by idiopathic CD4+ T lymphocytopenia. In contrast to previous reports, the isolate of *M. abscessus* subsp. *bolletii* presented intermediate resistance to clarithromycin and was susceptible to cefoxitin and imipenem.

## Introduction

Rapidly growing mycobacteria (RGM) are opportunistic pathogens in patients with underlying risk factors. These organisms are ubiquitous in soil and water and can cause skin, soft tissue, pulmonary, and central nervous system (CNS) infections and disseminated disease [1]. *Mycobacterium abscessus* subsp. *bolletii* is a newly recognized member of the RGM family, isolated from respiratory tract and cutaneous infections [2-7].

Idiopathic CD4+ T lymphocytopenia (ICL) is a rare syndrome, defined in 1992 as: (1) absolute CD4+ T lymphocyte level <300cells/μL or <20% of total lymphocytes at a minimum of two separate time points at least six weeks apart; (2) no serological evidence of human immunodeficiency virus (HIV) infection; (3) the absence of any defined immunodeficiency or therapy associated with depressed levels of CD4+ T cells [8,9]. The epidemiologic data do not suggest that the condition is caused by a transmissible agent and the exact incidence of the disease is variable according to different studies. Unlike HIV infection, the decrease in the CD4+ cell counts of patients with ICL is often slow or even absent over time, the number of CD8+ T cells is normal, and the immunoglobulin levels are either normal or slightly low. The pathogenesis of ICL may be multi-factorial. The diminished availability of stem cell precursors, the increased T cell apoptosis, the defective production of tumor necrosis factor (TNF)α and interferon (IFN)γ, the CD4+ T cell antibody production and impaired early biochemical events of the CD3-T cell receptor (TCR) pathway may be involved in the development of CD4+ T cell depletion in patients with ICL. ICL is typically diagnosed in middle age, usually after an opportunistic infection [9]. CD4 + T cells have a key role in the immunological response against mycobacterial infections and other parasitic and viral infections. Cryptococcal and nontuberculous mycobacterial infections are the major presenting opportunistic infections [10]. We describe a case of chronic disseminated infection caused by *M. abscessus* subsp. *bolletii* in a patient with ICL.

## Case presentation

Our patient was a 38-year-old previous healthy Sri Lankan man who had been living in Italy for two years. At admission only a history of allergic rhinitis was reported and corticosteroids intake was denied. He was diagnosed with a right pleural empyema and tubercular spondylitis. Ziehl-Neelsen staining of his pleural exudate yielded acid-fast bacilli and a biopsy of the pleura showed mycobacterial histopathological features.

Our patient was treated with an anti-tubercular treatment of isoniazid (300mg once a day, orally), ethambutol (400mg three times a day, orally), rifampicin (600mg once a day, orally) and pyrazinamide (500mg three times a day, orally) for one year.

**Figure 1:**
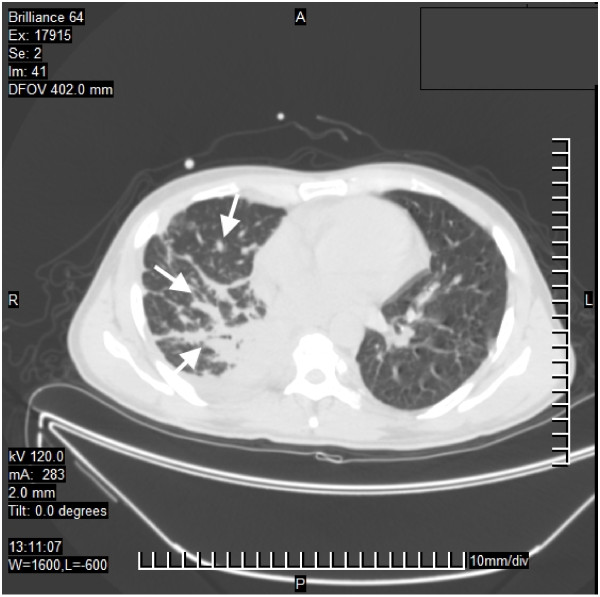
**Computed tomography image of lung infection by *Mycobacterium abscessus *subsp. *bolletii*.** The white arrows show parenchymal consolidation in both lungs, predominantly in the right side, and patchy ground-glass appearance with nodules.

After 15 months, our patient developed seizures and was admitted to an emergency room. Magnetic resonance imaging (MRI) scan of the brain revealed a nodular lesion in the right frontal lobe surrounded by peri-lesional edema. A thoracic computed tomography (CT) scan showed parenchymal consolidation in both lungs, predominantly in the right side (Figure 1). Acid-fast bacilli were detected on a Ziehl-Neelsen-stained sputum smear and in his bronchial aspirate. A polymerase chain reaction (PCR) investigation for *Mycobacterium tuberculosis* performed on a bronchial aspirate sample yielded a negative result. A culture of bronchial aspirate yielded nontuberculous mycobacteria. Our patient was moved to our Infectious Diseases Department at the University Hospital of Palermo.

On admission, our patient was afebrile. He complained of having had a cough for several weeks, sputum production and weight loss (5kg during the last two months). On chest auscultation, a reduction of vesicular breath sound was heard in the right lung base. On skin examination, painless mobile subcutaneous nodules, recently appeared, were seen on the frontal region, the scrotum and the left thigh. His abdominal, cardiac and neurological examination results were normal. He was on phenobarbital treatment.

Laboratory analyses showed a white blood cell count of 5.14 × 10^3^cells/μL, a hemoglobin level of 12.7g/dL, and a platelet count of 235 × 10^3^cells/μL. The total lymphocyte count was 580cells/μL and the CD4+ T lymphocyte level was 138cells/μL, corresponding to 23.9% of total lymphocytes. His serum immunoglobulin level was within normal limits.

The results of an HIV antibody test conducted on admission were negative, and again negative when the test was repeated two months later. Intra-dermal Mantoux testing results and serology tests for human T-lymphotropic virus (HTLV)1 and HTLV2 were also all negative. IgM and IgG anti-*Toxoplasma gondii* tests were negative, and cytomegalovirus (CMV) IgG were present. Our patient refused a lumbar puncture. *Pneumocystis jirovecii* was investigated using sputum samples and the results were negative. Therapy with anti-tubercular drugs (ethambutol 400mg three times a day, orally, isoniazid 300mg a day, orally and pyrazinamide 500mg three times a day, orally) had been maintained because of a history of tubercular pleurisy and spondylodiscitis. We started clarithromycin (500mg twice a day, orally) and rifabutin (450mg once a day, orally). Ziehl-Neelsen staining, culture and PCR tests for *M. tuberculosis* performed on three different sputum and urine samples all gave negative results. CD4+ T lymphocyte counts persisted at under 200cells/μL until discharge one month later.

Ultrasonography of the nodules revealed confined hyper-echoic areas with small hypo-echoic nodules and spotty blood flow signals in the subcutaneous cellular tissue. Testicular ultrasonography revealed diffuse enlargement and heterogeneous echo texture of the right epididymis, a small hypo-echoic nodule in an enlarged right testis, and a minimal hydrocele.

At discharge, the subcutaneous nodules had almost retreated but a chest radiograph revealed a new parenchymal consolidation area in the left lung. Our patient continued the abovementioned treatment until May 2008, when he arbitrarily stopped hospital follow-up and therapy.

Two years after diagnosis, our patient came back to our facility because of a new episode of seizures. Brain MRI scan findings were unmodified and a chest radiograph revealed multiple infiltrates involving both lungs. His CD4+ T lymphocyte count was 169cells/μL. Ziehl-Neelsen staining of a sputum specimen was positive for acid-fast bacilli, while PCR for *M. tuberculosis* was negative. While waiting for microbiological culture results, we started a treatment that could act against nontuberculous mycobacteria (clarithromycin 500mg twice a day, rifabutin 300mg once a day and ciprofloxacin 500mg twice a day). Cultures on both solid (Löwenstein–Jensen) and liquid (MGIT™ Becton Dickinson, Sparks, MD USA) media yielded the presence of acid-fast bacilli. The culture isolate was identified by analysis of its characteristics on culture (time for growth, colony morphology, pigment production, ability of the isolate to grow at various temperatures on 5% sheep blood agar and Löwenstein–Jensen slants) and by sequence analysis of its ribonucleic acid (RNA) polymerase β-subunit-encoding gene (*rpoB*) as *M. abscessus* subsp. *bolletii*[4]. In particular, after PCR amplification by using the consensus primers described by Adékambi *et al.* sequences corresponding to 752 bp of the *rpoB* gene were identified by similarity analysis with the sequences in the GenBank database by use of the Basic Local Alignment Search Tool (BLAST) software ( http://www.ncbi.nlm.nih.gov/BLAST). Anti-microbial drug susceptibility tests were performed using the methods described by Adékambi *et al*. [5]. The results are shown in the Table 1. The results of the anti-microbial susceptibility tests were interpreted according with the criteria proposed by the Clinical and Laboratory Standard Institute (CLSI, formerly NCCLS) and Brown-Elliot *et al*. [6,7].

**Table 1:** **Anti-microbial drug susceptibility test results for *Mycobacterium abscessus *** **subsp. *bolletii *** **isolate**

**Anti-microbial agent**	**Minimum inhibitory concentration (MIC) (μg/mL)**	**Interpretive criteria[**[7]**]**
Amikacin^a^	32	Intermediate
Amoxicillin clavulanate^b^	>256	Resistant
Cefotaxime^c^ (disk, 30μg)	>64	Resistant
Cefoxitin^b^	<8	Susceptible
Ciprofloxacin^a^	>16	Resistant
Clarithromycin^a^	16	Resistant
Cotrimoxazole^c^ (disk, 1.25/23.75μg)	>8	Resistant
Erythromycin^c^ (disk, 15μg)	>8	Resistant
Imipenem^b^	0.75	Susceptible
Levofloxacin^c^ (disk, 5μg)	>4	Resistant
Linezolid^c^ (disk, 30μg)	>8	Intermediate/Resistant
Minocycline^c^ (disk, 30μg)	<4	Susceptible
Ofloxacin^c^ (disk, 5μg)	>8	Resistant
Teicoplanin^b^	>256	Resistant
Tobramycin^c^ (disk, 10μg)	>8	Resistant
Tigecycline^b^	>256	Resistant
Vancomycin^b^	>256	Resistant

^a^Broth microdilution; ^b^Etest® (AB Biodisk) at 30°C for 72 hours incubation; ^c^disk diffusion.

Treatment was switched to azithromycin (500mg once a day, orally) and minocycline (100mg twice a day, orally). Because of the appearance of inguinal and axillary lymphadenopathy, a biopsy of inguinal lymph nodes was performed and this showed necrotizing granulomatous lymphadenitis. No acid-fast bacilli were seen on Ziehl-Neelsen staining. Three months after the first positive culture, *M. abscessus* subsp. *bolletii* was again isolated from sputum samples. Our patient is still on therapy and ambulatory follow-up and is in a good clinical condition. We did not perform an MRI because he refused any other diagnostic procedure.

## Discussion

*M. bolletii* is a nontuberculous mycobacteria recently reclassified as a subspecies of *M. abscessus* and renamed *M. abscessus *subsp. *bolletii*[2]. It has been described in respiratory tract infections in patients with coexisting broncho-pulmonary diseases and in subcutaneous infections from patients who had undergone mesotherapy [8,9,11].

RGM respiratory disease most often affects patients older than 60 years. Almost all patients younger than 40 years have one of the predisposing disorders [12,13]. The first case of disseminated *M. abscessus* subsp. *bolletii* infection in a young adult patient was described by Koh *et al.*[13]. Our patient was affected by ICL, a rare cause of immunodysfunction that, similar to HIV infection, causes a depletion of CD4+ T lymphocytes. ICL diagnosis was confirmed by persistent CD4+ T lymphocytes count under 300cells/μL in the absence of HIV infection and common variable immunodeficiency.

The clinical spectrum of ICL ranges from an asymptomatic laboratory abnormality to life-threatening complications. ICL is typically revealed by the emergence of opportunistic infections mainly nontuberculous mycobacteria and *Cryptococcus* spp. [14,15]. Disseminated mycobacterial infections have been rarely reported in patients affected by ICL [16-18]. This is the first report of *M. abscessus* subsp. *bolletii* infection in a patient affected by ICL. Our patient presented with a multi-focal spread of *M. abscessus* subsp. *bolletii* infection consisting in pulmonary, CNS, genital, lymphonodal, subcutaneous involvement. Pulmonary disease is an uncommon but clinically relevant entity caused by RGM, most often by the *M. abscessus* group [19].

CNS is a rare localization of RGM infection. The most common cause is dissemination from pulmonary infection [1]. The clinical presentation was dominated by the sudden appearance of seizures due to a frontal lobe nodular lesion. A cerebral localization of *M. abscessus* subsp. *bolletii* is highly probable due to the concomitant presence of chronic lung infection in a patient who is immunocompromised and the presented MRI findings.

Orchiepididymitis, lymphadenitis and subcutaneous nodules could be interpreted as other localizations presumably due to hematogenous spread from *M. abscessus* subsp. *bolletii* lung infection. Ultrasonographic and/or microscopic pictures were suggestive for mycobacterial infection [12,20,21].

As other RGM, *M. abscessus* subsp. *bolletii* is characterized by an inherent resistance to first-line anti-tuberculous drugs. In addition, it has been described as a multi-drug resistant mycobacterium, notably to clarithromycin but with intermediate resistance to amikacin [4]. In contrast, in our patient’s case the isolate of *M. abscessus* subsp. *bolletii* presented resistance to clarithromycin and was susceptible to cefoxitin, imipenem and minocycline (Table 1). It has been reported by Wallace *et al.* that the activity of tigecycline *in vitro* against several species of RGM was 11 times higher than other tetracyclines [21]. Conversely, the present *M. abscessus* subsp. *bolletii* isolate was resistant to tigecycline despite its susceptibility to minocycline. These differences could be due either to variability in antibiotic susceptibility within *M. abscessus* species or to the employ of different laboratory methods [22]. The therapeutic choice of azithromycin and minocycline was determined by our patient’s poor compliance and by its ability to cross the hematoencephalic barrier.

The natural course of our patient’s infection seems to be indolent even if the few descriptions of *M. abscessus* subsp. *bolletii* respiratory infection show high lethality [9]. However, the appropriateness of the therapeutic regimen could have also been negatively affected by some inconsistencies of the susceptibility testing approach.

The actual prognosis is difficult to establish because of his concomitant ICL, which continuously predisposes patients to various opportunistic infections.

## Conclusions

Accurate identification of RGM species is very important with regard to their different drug susceptibility patterns, clinical significance and prognosis [19]. The emergence of new multi-drug resistant species is a threat in consideration of the increasing number of patients with immune system impairment of different origin.

## Consent

Written informed consent was obtained from the patient for publication of this case report and any accompanying images. A copy of the written consent is available for review by the Editor-in-Chief of this journal*.*

## Competing interests

The authors declare that they have no competing interests.

## Authors’ contributions

LS, RR and LT analyzed and interpreted the data from our patient regarding clinical features, laboratory findings and computed tomography images. CM, CB and CC performed the microbiological analysis of the kidney samples. CC was a major contributor in writing the manuscript. All authors participated in analysis and interpretation of the data, and read and approved the final manuscript.
